# Methyl 3,4-dihydroxybenzoate alleviates oxidative damage in granulosa cells by activating Nrf2 antioxidant pathway

**DOI:** 10.1186/s13048-024-01412-5

**Published:** 2024-04-25

**Authors:** Shishi Li, Yuhang Fan, Chongyi Shu, Yier Zhou, Jing Shu

**Affiliations:** Department of Reproductive Endocrinology, Center for Reproductive Medicine, Zhejiang Provincial People’s Hospital, Affiliated People’s Hospital, Hangzhou Medical College, Hangzhou, 310000 People’s Republic of China

**Keywords:** Reactive oxygen species, Granulosa cells, Endometriosis, Drug therapy, Methyl 3,4-dihydroxybenzoate (MDHB)

## Abstract

Oxidative damage induced granulosa cells (GCs) apoptosis was considered as a significant cause of compromised follicle quality, antioxidants therapy has emerged as a potential method for improving endometriosis pregnancy outcomes. Here, we found that GCs from endometriosis patients show increased oxidative stress level. Methyl 3,4-dihydroxybenzoate (MDHB), a small molecule compound that is extracted from natural plants, reversed tert-butyl hydroperoxide (TBHP) induced GCs oxidative damage. Therefore, the aim of this study was to assess the protective effect of MDHB for GCs and its potential mechanisms. TUNEL staining and immunoblotting of cleaved caspase-3/7/9 showed MDHB attenuated TBHP induced GCs apoptosis. Mechanistically, MDHB treatment decreased cellular and mitochondria ROS production, improved the mitochondrial function by rescuing the mitochondrial membrane potential (MMP) and ATP production. Meanwhile, MDHB protein upregulated the expression of vital antioxidant transcriptional factor Nrf2 and antioxidant enzymes SOD1, NQO1 and GCLC to inhibited oxidative stress state, further beneficial to oocytes and embryos quality. Therefore, MDHB may represent a potential drug candidate in protecting granulosa cells in endometriosis, which can improve pregnancy outcomes for endometriosis-associated infertility.

**Supplementary Information:**

The online version contains supplementary material available at 10.1186/s13048-024-01412-5.

## Introduction

Endometriosis is a chronic estrogen-dependent inflammatory disorder with the presence of endometrial tissue outside the uterine cavity, including both the glandular epithelium and stroma [1]. It is one of the most common benign gynecological disorders, affecting 10% of all women of reproductive age, and up to 30 − 40% of women with infertility are affected by endometriosis [2]. The pathogenic mechanism of endometriosis infertility is complex. In addition to pelvic fibrosis, adhesion and distortion of anatomical structure caused by lesions, it is also related to the immune inflammatory response of pelvic cavity and uterus caused by repeated bleeding infiltration of ectopic endometrium. The altered follicular microenvironment in endometriosis patients also is a contributing factor to infertility.

Oxidative stress (OS) or the imbalance between reactive oxygen species (ROS) and antioxidants in the follicular microenvironment is an important factor that induces endometriosis-associated infertility and has been speculated to negatively affect folliculogenesis, oocyte maturation, ovulation and embryogenesis [3, 4]. Oxidative damage results in compromised follicles in endometriosis linked to fecundity decreases is wildly accepted [5, 6]. Follicle is the microenvironment for oocyte development, and granulosa cells (GCs) are the main cells to construct the microenvironment of follicle [7]. Granulosa cells are divided into parietal granular cells and cumulus granular cells, which not only regulate follicular fluid components and influence oocytes, but transzonal projections (TZPs) directly deliver nutrients to oocytes and transmit biological signals through gap junction across the zona projection(ZP) [8]. Granulosa cells from patients with endometriosis exhibited higher levels of oxidative stress and apoptosis markers [9]. There is oxidation-antioxidation imbalance in the follicular fluid of ovaries with ipsilateral endometriotic ovarian cyst [10], the affected granulosa cells showed endoplasmic reticulum stress, apoptosis and senescence [11]. Granulosa cell apoptosis not only causes follicular atrexia and lowered oocyte reserve, but also impacts mitochondrial function and DNA integrity, which reduces the quality of oocytes [12]. Therefore, improving the micro-environment for follicular development by correcting the oxidative stress state is expected to be a promising method to improve the pregnancy outcome in endometriosis [13].

Oxidative stress has been recognized as one of the main mediators of female infertility by causing various reproductive pathologies in females such as endometriosis, PCOS, preeclampsia, spontaneous abortion, and unexplained infertility. Antioxidant supplementation, such as Melatonin and Resveratrol has been shown to exert beneficial effects on endometriosis [11, 14–16]. At present, most treatments for endometriosis focus on inhibiting focal angiogenesis and relieving pain symptoms, but few studies have reported on improving the environmental oxidation level of endometriosis follicles and improving oocyte quality [3]. We first focus on Methyl 3,4-dihydroxybenzoate (MDHB, C8H8O4, MW: 168.15) a small molecule compound that is extracted from East Asian Tang Materia, Malan and other natural plants [17]. Previous studies have demonstrated that MDHB has neurotrophic effects [18], anti-apoptotic effects induced by Aβ25–35 [19]as well as the lifespan extension effects on C. elegans [20]. MDHB has antioxidant and anti-inflammatory effects on bone marrow monocytes [4] and SH-SY5Y Cells [21]. However, the effects of MDHB on granulosa cells remain unclear.

In the present study, we found that cumulus granulosa cells in endometriosis patient have excessive oxidative stress. MDHB has anti-apoptotic and anti-oxidative effects in human ovarian granulosa cells-derived cell line (KGN) induced by tert-butyl hydroperoxide (TBHP). Furthermore, we found MDHB inhibited oxidative stress by promoting Nrf2-mediated antioxidative activity, reduced oxidative stress damage of granular cells, which is beneficial to improve the quality of oocytes and embryos.

## Materials and methods

### Isolation and culture of human granulosa cells

Our study was approved by the Ethical Review Board of Zhejiang Provincial People’s Hospital. Informed consent was obtained from each patient. Forty-one Han population patients seen for infertility at the Reproductive Medicine Center of Zhejiang Provincial People’s Hospital were included. The exclusion criteria were chromosomal abnormalities in any partner of a couple. The patients were divided into two groups. The control group (*n* = 20) patients include tubal infertility or male factor infertility, and another group women with endometriosis (*n* = 21) had visible peritoneal endometriotic lesions at laparoscopy. Clinical information on endometriosis and control patients was summarized in Table S1. GCs were mechanically removed by cutting the cumulus layer of each leading oocyte and washing twice in phosphate buffer saline (PBS) followed by centrifugation (1000 rpm for 5 min). GCs were digested with hyaluronidase(90,101, FUJIFILM IrvineScientific, Japan)for 5–10 min and cultured on 24-well plates in DMEM/ Ham’s F12 (Hyclone, Logan, UT) supplemented with 10% FBS and antibiotics (100 IU/mL penicillin and 100 µg/mL streptomycin obtained from Beyotime). The cells were cultured for 24 h and then used in the experiment.

### Human granulosa-like tumor cell line, KGN culture

The human ovarian granulosa-like tumor cell line (KGN), were purchased from the BeNa Culture Collection (BNCC337610, Beijing, China). KGN cells were cultured in DMEM/ Ham’s F12 (Hyclone, Logan, UT) supplemented with 10% FBS and antibiotics (100 IU/mL penicillin and 100 µg/mL streptomycin obtained from Beyotime). Cells were cultured in a 5% CO_2_ atmosphere at 37℃. When the cells grew to a density of 80%, the experiment was carried out by passage or planted.

### Cell viability assay

1 × 10^4^ KGN cells were planted in 96-well plates and incubated in various concentrations of MDHB (Selleck, USA) 0, 0.625, 1.25, 2.5, 5, 10 and 20 µM for 24–48 h in culture medium with serum. TBHP (Sigma, Germany) at 100 µM was used to induce oxidative stress. Cells were treated with 100 µM TBHP or without TBHP, treated by various concentrations of MDHB (0, 2.5, 5, 10 and 20 µM) for 24 h. Then, cell viability was determined by using the Cell Counting Kit-8 (YEASEN, China) assay following the manufacturer’s protocol.

### RNAs extraction and quantitative real-time polymerase chain reaction (qPCR) analysis

1 × 10^5^ cells were used for RNAs extraction and total RNAs were extracted using an Ultrapure RNA Kit (CW0581, CWBIO, China) according to the manufacturer’s instruction and RNAs quantity was measured by NanoDrop 2000. The cDNAs were synthesized using HiFi-Script cDNA Synthesis Kit (CW2569, CWBIO, China). qPCR assays were performed using the UltraSYBR Mixture (CW0957, CWBIO, China) according to the manufacturer’s instructions in triplicate. The relative genes expression levels were normalized to β-actin. The primer sequences used in this study are listed in Table 1.

**Table 1:** qPCR primer sequences

Gene	Sequence
SOD1	F: 5’-GGTGGGCCAAAGGATGAAGAG-3’
	R: 5’-CCACAAGCCAAACGACTTCC-3’
NQO1	F: 5’-GAAGAGCACTGATCGTACTGGC-3’
	R: 5’-GGATACTGAAAGTTCGCAGGG-3’
GCLC	F: 5’-GGAGGAAACCAAGCGCCAT-3’
	R: 5’-CTTGACGGCGTGGTAGATGT-3’
Nrf2	F: 5’-TCAGCGACGGAAAGAGTATGA-3’
	R: 5’-CCACTGGTTTCTGACTGGATGT-3’
β-actin	F: 5’-CATGTACGTTGCTATCCAGGC-3’
	R: 5’-CTCCTTAATGTCACGCACGAT-3’

### Detection of cellular ATP levels

5 × 10^4^ KGN cells were planted in 24-well plates and exposed to 100 µM TBHP or without TBHP and treated by 20µM MDHB for 24 h. Then the cells were collected, ATP levels in GC lysates were measured using a luminometer according to the manufacturer’s instructions (Beyotime Biotechnology, China).

### Assessment of oxidative stress

The intracellular levels of ROS in KGN were measured by 2′,7′-Dichlorofluorescein diacetate (DCHF-DA) according to the manufacturer’s instructions (Beyotime Biotechnology, China). In brief, The KGN cells were incubated in PBS containing 10 µM DCHF-DA at 37 ℃ for 15 min under dark. After being washed three times with PBS, Then observed immediately under an fluorescence microscopy (Nikon, Japan) at an excitation wavelength of 490 nm and an emission wavelength of 525 nm.

### Mitosox staining

In order to detect the level of superoxide inside the mitochondria of KGN cells, we use MitoSOX Red Mitochondrial Superoxide Indicator (MCE, USA)0.5 × 10^4^ KGN cells were planted in 24-well plates and exposed to 100 µM TBHP or without TBHP and treated by 20µM MDHB for 24 h. Wash the stimulated KGN cells twice with pre-warmed PBS, add 125uL MitoSOX (10 µM) staining working solution, incubate at 37 °C for 30 min in the dark, then add PBS to wash three times, and cover the KGN cells with 500uL DMEM medium per well. Then observed immediately under a fluorescence microscopy microscope (Nikon) at an excitation wavelength of 561 nm and an emission wavelength of 590 nm.

### Detection of mitochondrial membrane potential (MMP)

Mitochondrial membrane potential (MMP) in KGN was measured by staining with 5,6,6-Dichloro-1,1,3,3-tetraethyl-imidacarbocyanineiodide (JC-1) according the manufacturer’s instructions (Beyotime Biotechnology, China). In brief, 5 × 10^4^ KGN cells were planted in 24-well plates and exposed to 100 µM TBHP or without TBHP and treated by 20µM MDHB for 24 h. KGN cells were incubated in pre-heated DMEM medium containing 1 µM JC-1 for 15 min at 37 °C in the dark. After incubation, KGN were washed twice with PBS and observed under a Fluorescence microscopy (Nikon, Japan) equipped with laser source at excitation/emission wavelength of 488/510 nm (to visualize green JC-1 monomers) or 561/590 nm (to visualize red JC-1 aggregate). Image J software was used to quantitatively analyze the fluorescence intensity of acquired images with JC-1 staining. MMP was calculated as a ratio of the fluorescence intensity at 590/510 nm.

### Detection of apoptosis

Cell apoptotic indices in KGN as measured by the DNA fragmentation was determined using terminal deoxynucleotidyl transferase dUTP nick end labeling (TUNEL) according to the manufacturer’s instructions (Beyotime Biotechnology, China). Briefly, 5 × 10^4^ KGN cells were planted in 24-well plates and exposed to 100 µM TBHP or without TBHP and treated by 20µM MDHB for 24 h, then the cells were fixed in 4% paraformaldehyde in PBS for 30 min, followed by permeabilization in 0.2% Triton X-100 in PBS for 5 min. Then wash with PBS 3 times and incubate 50µL TUNEL working solution per well for 1 h at 37 °C. Finally observe with a fluorescence microscope.

### Western blot

The proteins were collected using RIPA lysis buffer (Bio-Rad, USA) containing protease inhibitor and were quantified using Pierce™ Pierce BCA Protein Assay Kit (Thermo Fisher Scientific, Waltham, MA). Proteins were separated by electrophoresis on a 10% SDS polyacrylamide gel and then transferred onto polyvinylidene fluoride (PVDF) membranes (Millipore, USA). Membranes were incubated in 5% skimmed milk for blocking. Then the membranes were incubated overnight with primary antibodies against β-actin (Beyotime Biotechnology, China), Casp3 (CST, USA), C-Casp3 (CST, USA), Casp7 (CST, USA), C-Casp7 (CST, USA), Casp9 (CST, USA), C-Casp9 (CST, USA), Nrf2 (Abcam, USA) at 4℃. After washed three times in TBST, the membranes were invubated with Hrp-conjucated secondary antibody for 45 min in room temperature. Protein bands were detected by staining with enhanced chemiluminescence (ECL) (Bio-Rad, USA). The results were analysed by Image J software.

### Animal handling

The mouse model of endometriosis was established according to the published paper [22]. In brief, both the donor and recipient were C57BL/6 mice (4–6 weeks, 19–22 g). Uterus of donor mice stimulated by estradiol (0.2mL/ piece) were cut into 4 × 4 mm endometrial tissue slices in PBS solution at 4℃ for use. After anesthesia, the donor endometrial tissues were sewed to the peritoneal wall near the recipient mice ovary, and the ovarian surface sheath was removed with PBS. After the abdominal cavity was closed, 200uL PBS was injected intraperitoneally to prevent adhesion, and estradiol was given to promote the growth of the graft intima. The recipient mice were given continuous antibiotics 2 days after surgery to prevent infection.

Female mice with ovarian endometriosis model were divided into 3 groups: control mice + PBS group (con, *n* = 4), endometriosis mice + PBS group (EM, *n* = 4), endometriosis mice + 10 mg/kg MDHB group (EM + MDHB, *n* = 4). The endometriosis mouse was given 10 mg/kg MDHB intervention two weeks after modeling and once every four days for 4 weeks according to previous study [17]. All mice were administrated by intraperitoneal injection.

### Oocyte collection and in vitro fertilization

Female mice were superovulated by intraperitoneal injection with 10 IU of pregnant-mare serum gonadotropin (NSHF, China), followed by treatment with 10 IU of human chorionic gonadotropin (HCG, NSHF, China) 48 h later, and then were euthanized 15 h after HCG treatment to collect oocytes. The oviducts from stimulating ovulation mice were transferred to a clean dish with MOPS medium (Vitrolife, Sweden). Oocytes were released from the oviduct under a thermal platform stereomicroscope and washed twice with MPOS containing 10% v/v serum substitute (Irvine Scientific, USA) and transferred into IVF medium (Vitrolife, Sweden). We have got a total of 56 oocytes and divided into 3 groups: control group (con, *n* = 19), endometriosis group (EM, *n* = 18), endometriosis + MDHB group (EM + MDHB, *n* = 19). At the same time, sperm from the tail of epididymis was obtained from mature male mice and cultured in IVF medium for 30 min. And then sperms were added to the oocytes for in vitro fertilization 2 h later. Detailed information and procedures are described in published study [23].

### Embryo culture

Two-cell embryos were transferred into G1 plus medium (Vitrolife, Sweden). The embryos were then washed and cultured in G2 plus medium (Vitrolife, Sweden) for another 48 h to reach the blastocyst stage. The scoring and collection of embryos at various developmental stages were based on the embryo morphology. approximately as follows, following HCG injection: 2-cell, 36 h ; cleavage, 60 h ; morula, 72 h; and blastocyst, 96 h.

All experimental protocols concerning the use and care of mice in this study were reviewed and approved by the Institutional Animal Ethics Committee of Hangzhou Medical College (approval number 20,190,194).

### Statistical analysis

All experiments were conducted at least in triplicate. Data analyses were conducted using SPSS 19.0 (SPSS, Chicago, IL, USA). Statistical comparison between two groups was carried out using the unpaired Student’s t-test after confirming the normal distribution of the data by One-Sample Kolmogorov-Smirnov Test or Mann-Whitney U Test. One-way or two-way ANOVA were used for multiple comparisons. * *p* < 0.05 was considered statistically significant.

## Results

### GCs from endometriosis patients show increased oxidative stress

**Fig. 1:**
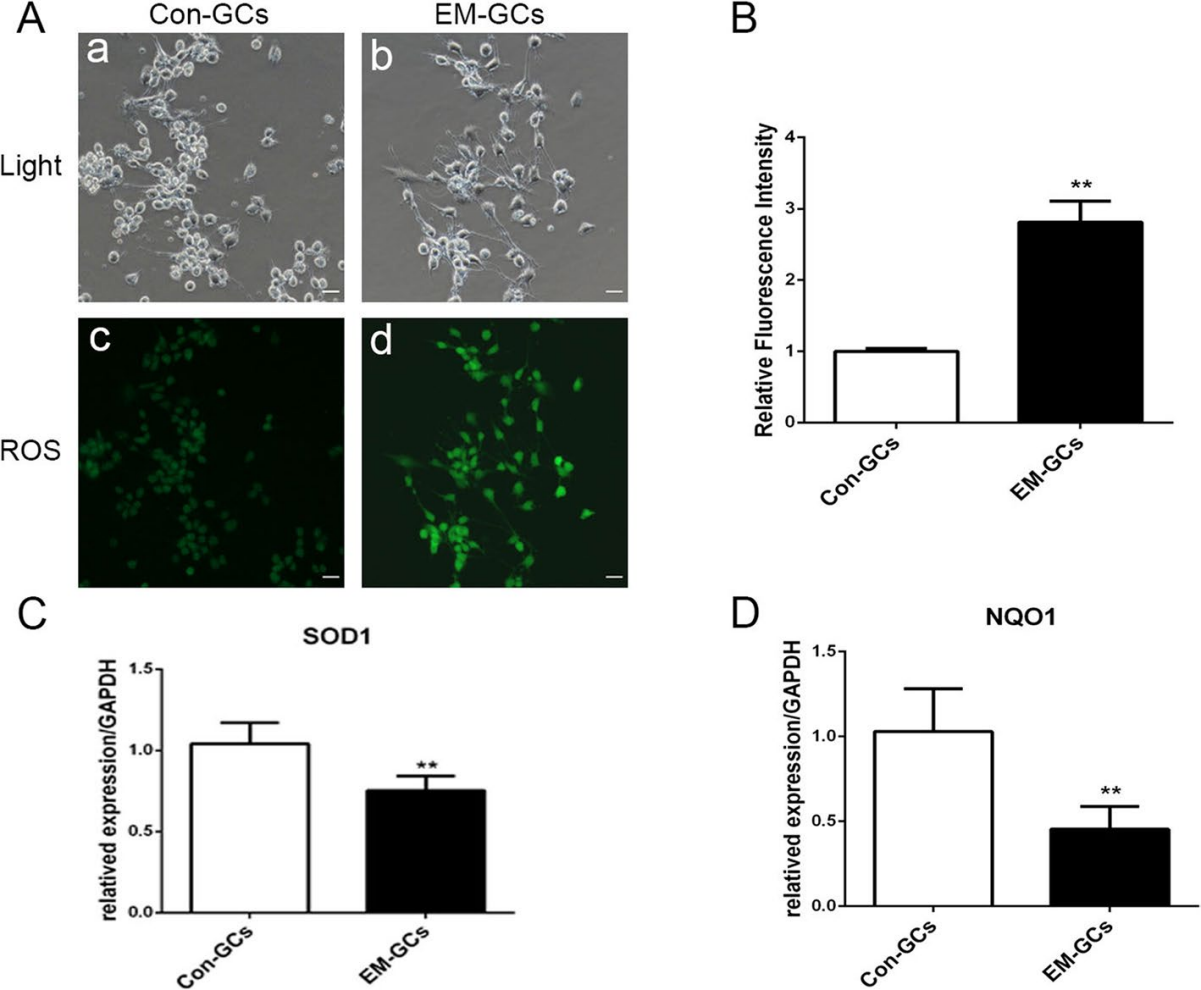
GCs from endometriosis patients show increased oxidative stress. **A**, **B** Granulosa cells were isolated from endometriosis (EM-GCs) and non-endometriosis patients (Con-GCs), ROS level was detected by DCFH-DA dye. **C**, **D** mRNA expression level of SOD1 and NQO1 were detected in Con-GCs and EM-GCs groups. All data were presented as the Means ± SD, **P < 0.01

To investigate whether the level of oxidative stress in granulosa cells of endometriosis patients is changed, we first examined ROS levels in fresh collected in vitro fertilization (IVF) patient GCs by the oxidation-sensitive dye DCFH-DA. As shown in Fig. 1A, B, we found intracellular ROS in GCs from endometriosis patients (EM-GCs) was significantly increased compared with that in control GCs. The antioxidant-related genes superoxide dismutase 1 (SOD1) and NAD(P)H quinone dehydrogenase 1 (NQO1) were significantly reduced in these GCs compared with control GCs (Fig. 1C, D). These results confirmed GCs from endometriosis patients show increased oxidative stress.

### Protective effect of MDHB on TBHP treated KGN cells

**Fig. 2:**
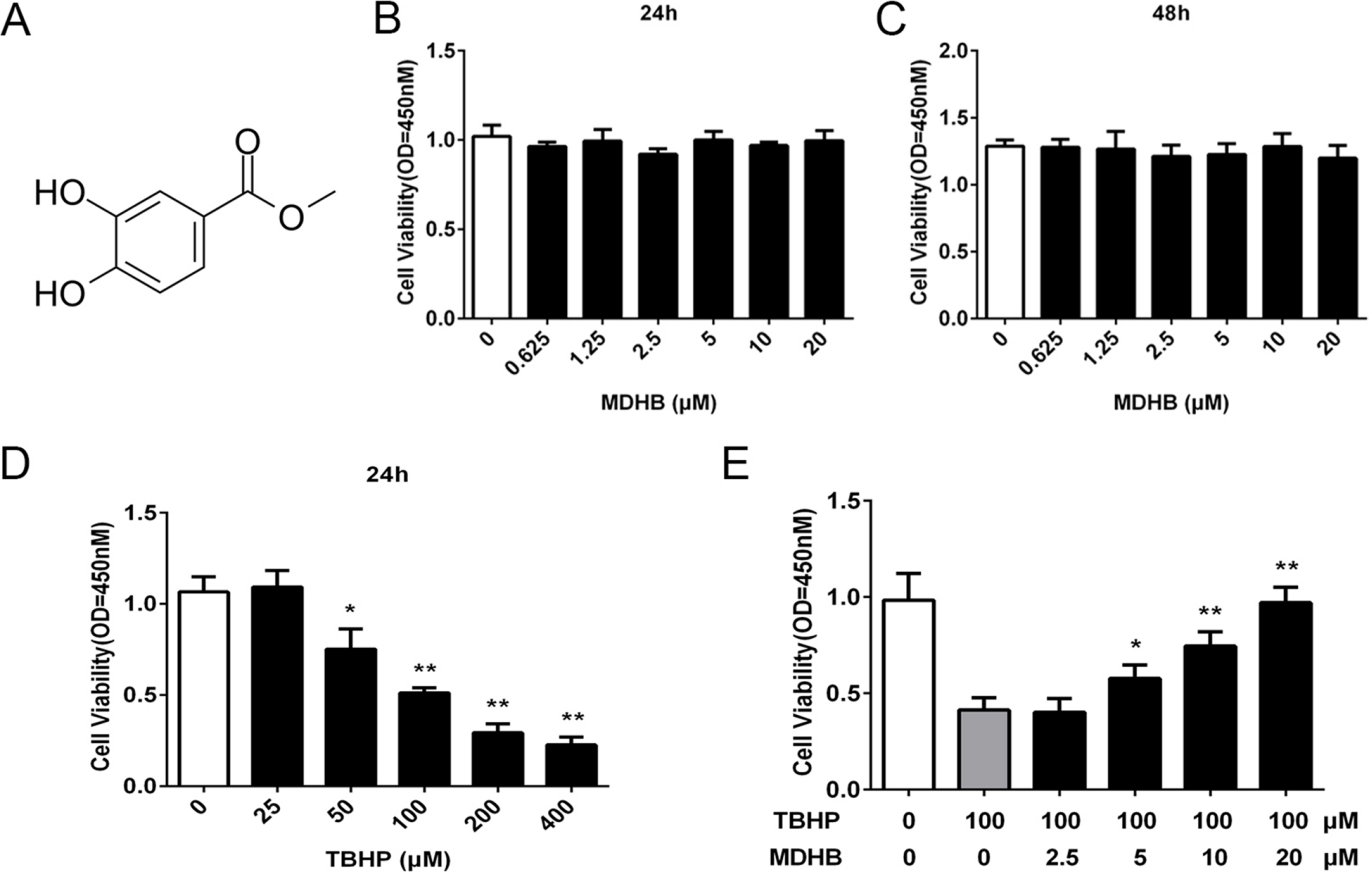
Protective effect of MDHB on TBHP treated KGN cells. **A** The structural formula of Methyl 3,4-dihydroxybenzoate (MDHB). **B**, **C** The effects of MDHB on KGN cell viability under different concentrations by 24–48 h treatment. **D** The effects of TBHP on KGN cell viability under different concentrations by 24 h treatment. **E** MDHB treatment rescued TBHP induced KGN cell death under different concentrations. All data were presented as the Means ± SD, *P < 0.05, **P < 0.01

To investigate the potential effect of MDHB, as shown the structural formula in Fig. 2A, on granulosa cells, we first detect the possible cytotoxicity of MDHB on the cell viability. CCK-8 assays were carried out and the results demonstrated that compared with control groups, treatment with MDHB at concentrations below 20µM for either 24 h (Fig. 2B) or 48 h (Fig. 2C) did not show any significant cytotoxic effect. TBHP is commonly used to induce oxidative stress, we then detected the effects of TBHP on the viability of GC line KGN. Compared with the control group, the viability of KGN was reduced with increasing concentration of TBHP, the IC50 value of TBHP for KGN was 100µM for 24 h treatment, as CCK-8 revealed in Fig. 2D. Therefore, the TBHP concentration of 100µM was utilized in the subsequent experiments. To test the protective effects of MDHB on TBHP induced cell death, the cells were treated with different concentrations of MDHB for 24 h while being exposed to TBHP. As shown in Fig. 2E, MDHB showed protective effect at concentrations of 5, 10, 20µM. Above findings suggested that the TBHP induced cytotoxicity was reversed by MDHB.

### MDHB attenuates TBHP induced KGN cells apoptosis

**Fig. 3:**
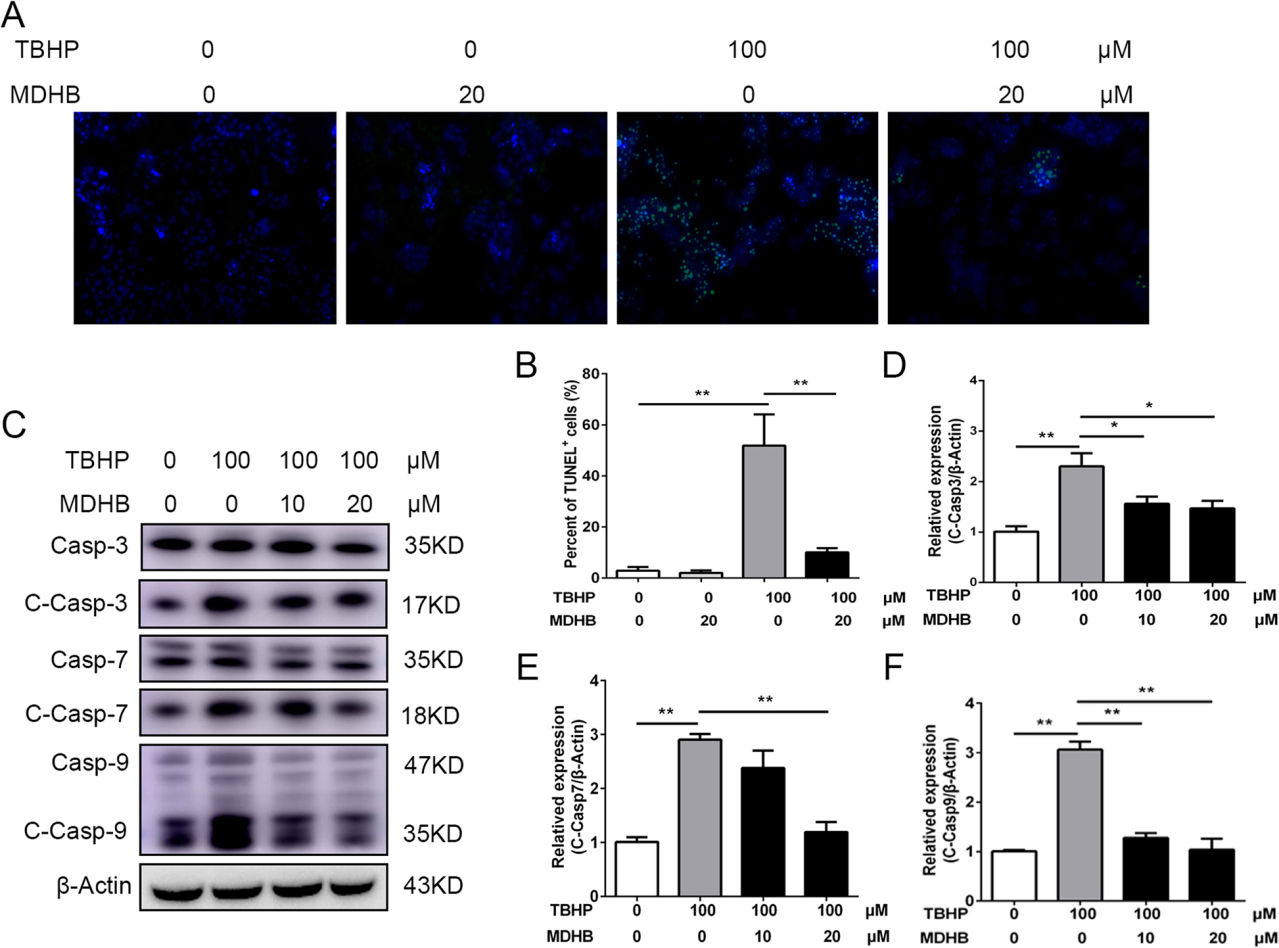
MDHB attenuates TBHP induced KGN cells apoptosis. **A**, **B** TUNEL staining was performed to detected the effect of MDHB on TBHP induced GCs apoptosis. Representative immunofluorescence images of TUNEL (green) and nuclei labeled with DAPI. **C-F** Immunoblots showing the expression of Casp-3, C-Casp-3, Casp7, C-Casp-7, Casp-9 and C-Casp-9 in KGN cells treated with TBHP or MDHB (10, 20µM). Quantification of the protein levels relative to β-actin. All data were presented as the Means ± SD, *P < 0.05, **P < 0.01

GCs apoptosis was one of the main factors to cause infertility in endometriosis, we detected the effect of MDHB on TBHP induced apoptosis in GCs. TUNEL staining showed that the percentage of apoptotic KGN exposed to 100µM for 24 h was significant higher than that in control group. Importantly, treatment with MDHB significantly alleviated the increased apoptosis induced by TBHP (Fig. 3A, B). Consistent with the TUNEL staining result, Western blot analysis demonstrated considerably increased levels of apoptosis-associated proteins such as cleaved caspase-9 (C-Casp-9), cleaved caspase-7 (C-Casp-7) and cleaved caspase-3 (C-Casp-3) following treatment with TBHP. However, these were suppressed by MDHB treatment (Fig. 3C-F).

### MDHB protects KGN cells against TBHP‑induced oxidative stress

**Fig. 4:**
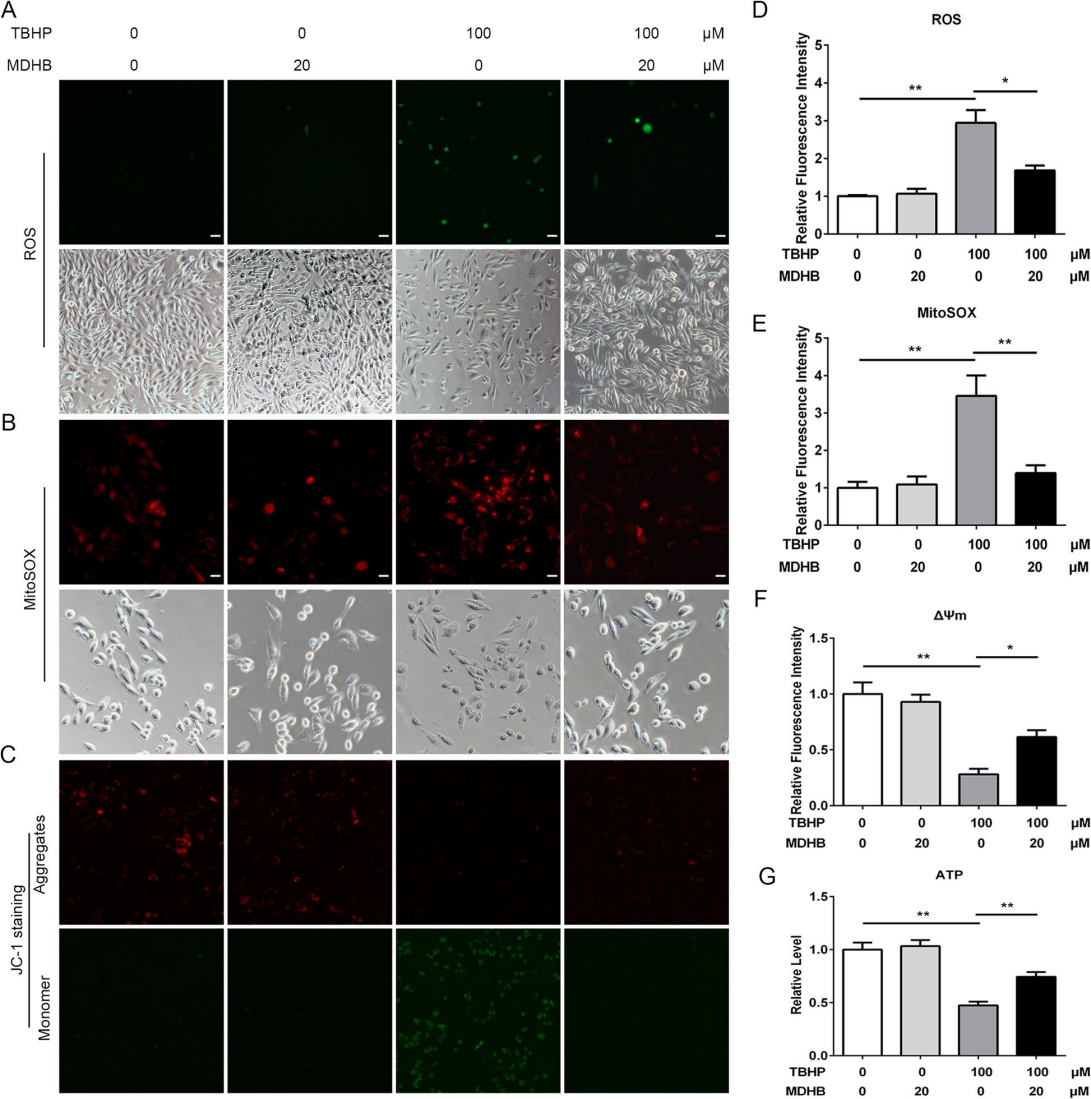
MDHB protects KGN cells against TBHP‑induced oxidative stress. **A** ROS staining (green) were performed to detected the protective effects of MDHB on TBHP‑induced oxidative stress in KGN cells. **B** MitoSOX staining (red) were performed to detected the protective effects of MDHB on TBHP‑induced oxidative stress in KGN cells. **C** JC-1 staining was detected for mitochondrial membrane potential, where green represents JC-1 monomers indicating low potential, and red represents JC-1 aggregates indicating high potential. **D-F** The analysis of relative fluorescence intensity for ROS, MitoSOX and JC-1 staining. **G** The effects of TBHP or MDHB treatment on ATP production. All data were presented as the Means ± SD, *P < 0.05, **P < 0.01

Previous studies have reported that oxidative damage caused by the excessive generation of ROS activates intracellular signaling cascades and leads to GCs apoptosis thus promoting infertility in endometriosis. Therefore, here we wondered whether MDHB protected GCs against TBHP‑induced oxidative stress. The level of oxidative stress in KGN was determined by measuring ROS using the oxidation-sensitive dye DCFH-DA and MitoSOX Red. We found that TBHP exposure significantly increased intracellular and mitochondrial ROS production in KGN, and they were both significantly suppressed by MDHB treatment (Fig. 4A-E). Excessive ROS is thought to hamper mitochondria function and lead to mitochondrial membrane potential (MMP) reduction and insufficient ATP supply, which are frequently detected in damaged GCs. We thus examined MMP in KGN using JC-1 staining. As shown in Fig. 4C, immunofluorescence showed stronger green JC-1 monomer signal and weaker red JC-1 aggregate signal in TBHP group compared with control group, while MDHB treatment rescued the reduction of MMP (Fig. 4F). Also, TBHP induced ATP reduction was reversed by MDHB treatment (Fig. 4G). All these findings indicated that TBHP-mediated oxidative stress and mitochondrial damage were rescued by MDHB.

### MDHB inhibited oxidative stress by promoting Nrf2-mediated antioxidative activity in KGN cells

**Fig. 5:**
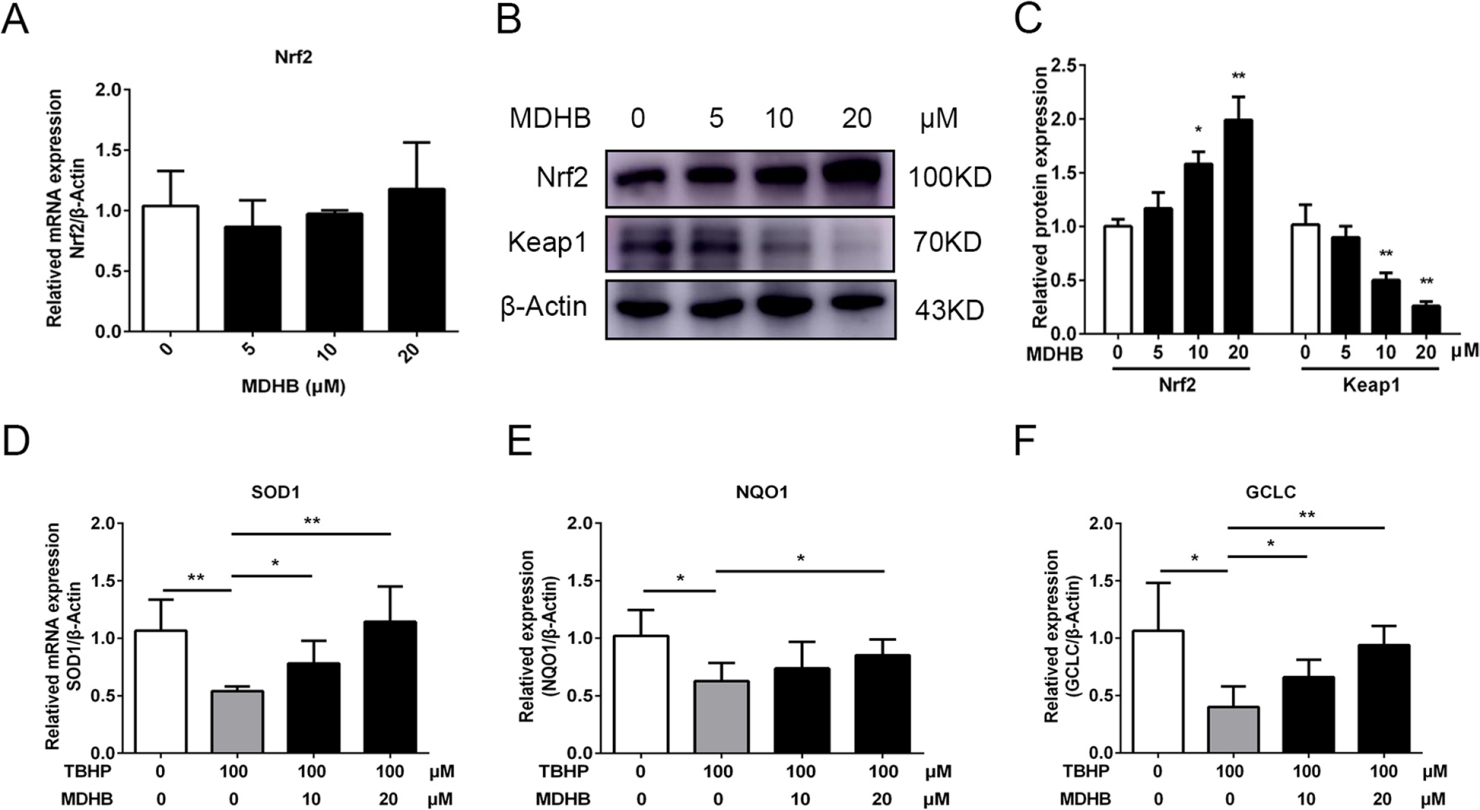
MDHB inhibited oxidative stress by promoting Nrf2-mediated antioxidative activity in KGN cells. **A** The effect of MDHB (0, 5, 10, 20µM) on Nrf2 gene expression by qPCR analysis. **B**, **C** Immunoblots showed the expression of Nrf2 and Keap1 in KGN cells treated with MDHB (0, 5, 10, 20µM). Quantification of the protein levels relative to β-actin. **D-F** qPCR analysis showed the expression of SOD1, NQO1 and GCLC in KGN cells treated with TBHP or MDHB (10, 20µM). All data were presented as the Means ± SD, *P < 0.05, **P < 0.01

For Nrf2 is a key transcription factor that promotes the gene expression of antioxidant enzymes such as SOD1, NQO1 and GCLC to against oxidative stress, we hypothesized that the protective effects of MDHB on oxidative stress and mitochondrial damage is associated with the Nrf2 pathway. To this end, we detected the expression of Nrf2 by treatment with MDHB at concentrations of 5, 10, 20µM for 24 h. Intriguingly, MDHB treatment significant upregulated Nrf2 protein level and decreased Keap1 protein level (Fig. 5B, C), but showed no significant effect on the Nrf2 mRNA expression (Fig. 5A), which suggested MDHB regulated Nrf2 expression at protein post-translational level. Next, downstream target genes of Nrf2, including SOD1, NQO1 and GCLC were detected to determine whether Nrf2 signaling was activated. As expect, the expression of SOD1, NQO1 and GCLC were upregulated in KGN after MDHB treatment (Fig. 5D-F). These results indicated that MDHB treatment contributes to activate Nrf2 signaling.

### MDHB improves embryo quality of endometriosis mice

**Fig. 6:**
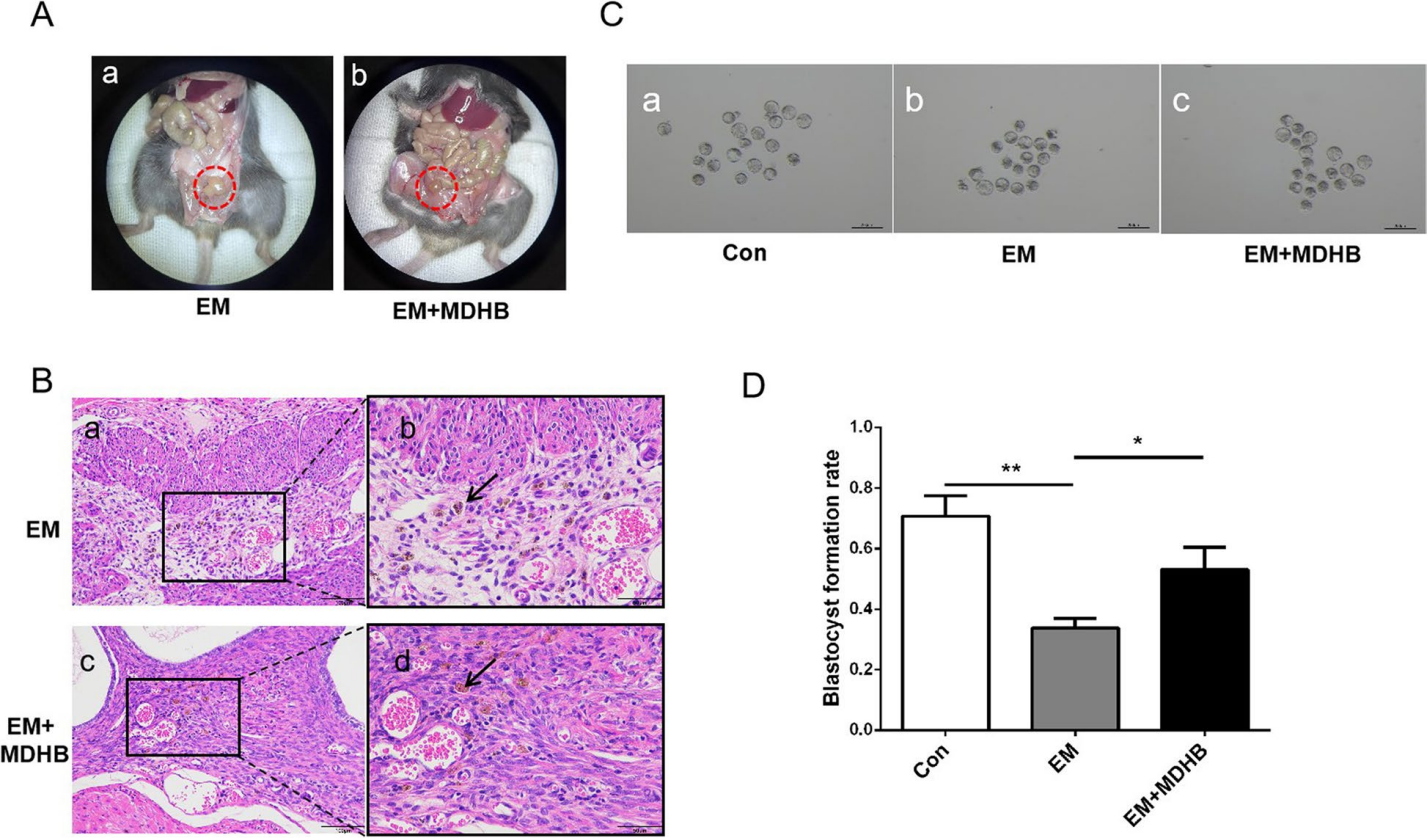
MDHB improves embryo quality of endometriosis mice. **A** Upper panel showing representative visible lesions within the peritoneal cavity of an endometriosis mice (EM) and an MDHB treated endometriosis mice (EM + MDHB) six weeks after surgery. **B** Histology confirmed endometriosis. The cystic lesions of EM and EM + MDHB group consisted of single-layered epithelium and underlying stoma with hemosiderin accumulation (black arrows), Scale bar = 100 μm for ×200 images. Scale bar = 50 μm for ×400 images. **C** The morphology of mouse embryos in blastocyst stage from control(*n* = 19), EM(*n* = 18) and EM + MDHB(*n* = 19) group (scale bar = 200 μm). **D** The blastocyst formation rate of control, EM and EM + MDHB group. All data were presented as the Means ± SD, ** *P* < 0.01, *P < 0.05

In order to verify that MDHB can also work in vivo for endometriosis. We established the mouse model of endometriosis and treated with MDHB for 4 weeks. The result showed in Fig. 6A, the cystic lesions contained clear light-yellow fluid as observed in EM group and were significant alleviated in EM + MDHB group (Fig. 6Aa-b). Histological examination demonstrated the cystic lesions as endometriosis. Cystic lesions of the EM and EM + MDHB group consisted with single-layered columnar epithelium without cilia, concomitant with underlying stromal tissue (Fig. 6Ba and c). Of note, the endometriotic stroma of model contained hemosiderin granules (Fig. 6Bb and d), which is often recognized in the human endometriotic lesion. Then, we superovulated mice and collected oocytes for in vitro fertilization, and observed the embryo morphology at blastocyst stage. The results showed that EM group had a significantly lower blastocyst rate than the control group, but the inhibitory effect was rescued in EM + MDHB group (Fig. 6C and D). Our results imply that MDHB reduces the oxidative stress level of granulosa cells and improves the quality of oocytes, thus facilitating blastocyst formation.

## Discussion

Oxidative stress induced apoptosis is a key pathogenesis of many diseases. Previous studies have shown that oxidative stress in the follicular microenvironment is an important factor that leads to infertility in endometriosis and has been speculated to hamper GCs function, folliculogenesis, and oocyte maturation [24]. Oxidative damage induced GCs apoptosis was considered as a significant cause of compromised follicle quality and adverse outcomes of assisted reproductive technology (ART) in endometriosis [25]. Thus, antioxidants therapy has emerged as a potential method for improving pregnancy outcomes.

Plants are the important resources of natural medicament, small-molecule compound extracted from natural plant has showed great value in clinic, such as salicylic acid, artemisinin, etc [26]. Methyl 3,4-dihydroxybenzoate (MDHB) is a major metabolite of polyphenols found in tea, East Asian Tang Materia, and other natural plants. Studies have reported that MDHB has effects of neuroprotection, anti-inflammatory, and antioxidation, which showed potential for diseases treatment. In the present study, we explored the possible protective role of MDHB in GCs.

We first detected the level of oxidative stress in granulosa cells from endometriosis patients and confirmed it was increased as others have reported. For TBHP-induced oxidative stress is a well-established common model for oxidative injury, we investigated the effect of MDHB in TBHP-treated GCs, and found MDHB protected GCs viability in a dose-dependent manner. Apoptosis was one of the main outcomes of cell oxidative damage, we found GCs apoptosis was significantly suppressed by MDHB treatment as showed by TUNEL staining and apoptosis-associated proteins detection.

Mitochondria are the center of energy and metabolism in eucaryotic cells and also are the vital cell organs in the process of transmitting apoptosis signs [27]. The pathological changes of mitochondrial-related dysfunctions are the main signals to initiation of apoptosis, including accumulation of ROS, reduction of MMP and decrease of ATP generation [28]. We wondered whether MDHB inhibited GCs apoptosis by scavenging ROS and protecting mitochondria function. As expected, we found MDHB treatment prominently reduced intracellular and mitochondrial ROS production, improved the mitochondrial function as showed by the rescued MMP and elevated ATP production. Lin et al.’s found melatonin administration rescued OS-enhanced ER stress, cellular senescence, and MMP and ATP abnormities of endometriosis GCs in an endometriosis mouse model [11]. Two recent studies demonstrated that Silymarin extract reduces the total serum anti-oxidant activity and induces the regression of endometriotic lesions in a rat model [29, 30]. Vitamins E and C administration decreased the levels of the oxidative stress marker myeloperoxidase (MPO) in follicular fluid from patients with severe endometriosis [31]. These previous research suggested that antioxidant supplementation may be a promising adjuvant therapy to reverse fertility decline in endometriosis.

Nuclear factor-erythroid 2 related factor 2 (Nrf2) is a crucial redox-related transcription factor for attenuating oxidative stress-associated pathological processes [32]. The ubiquitin-mediated proteasomal degradation is crucial to Nrf2 protein stability. Under normal physiological conditions, Kelch-like ECH-associated protein 1 (Keap1) binds to Nrf2 to promote the formation of a BCR (BTB-CUL3-RBX1) E3 ubiquitin ligase complex, which leads to proteasomal degradation of Nrf2. In response to oxidative stress, different electrophile metabolites trigger non-enzymatic covalent modifications of highly reactive cysteine residues in KEAP1, leading to inactivate the ubiquitin ligase activity of the BCR (KEAP1) complex, which inhibited Nrf2 degradation and promoted its nuclear translocation [33]. The nuclear localized Nrf2 could bind with antioxidant response element (ARE) fragments on DNA to positively regulate the expression of numerous antioxidant genes and phase II detoxifying enzymes, such as HO1, SOD1, NQO1, GCLC, and TrxR1 [34].

A number of natural compounds and metabolites were reported to exist antioxidative activities by activating Nrf2 signaling, such as catechin, which have been showed potential therapeutic effects on many diseases [35]. There have been reported MDHB promotes the expression of Nrf2 by reducing ubiquitination-induced proteasomal degradation of Nrf2 and reducing ROS levels, leading to inhibition of the activation of the MAPK and NF-κB pathways [17]. We speculated MDHB suppressed ROS production and apoptosis by regulating Nrf2 expression. Exactly, we found MDHB treatment upregulated Nrf2 protein level in dose-dependent manner and promoted downstream target genes expression including SOD1, NQO1 and GCLC. In addition, we noticed that MDHB did not affect Nrf2 gene expression, which implied MDHB influenced Nrf2 expression at post-translational modification level. Therefore, the mechanism would be explored in our further study.

In conclusion, this study confirmed that increased oxidative stress level in GCs isolated from patients with endometriosis. MDHB was a protective antioxidant against oxidative damage to GCs via the elimination of ROS and inhibited oxidative-stress induced apotosis. The underlying mechanism by which MDHB presented such effects involved the regulation of the protein of Nrf2.

In our current study, we just proved that GCs from endometriosis had higher ROS levels and higher expression of numerous antioxidant genes compared with control GCs, more precise signaling pathways should be explored in the future.Although our results suggest that MDHB may represent a potential drug candidate in protecting granulosa cells in endometriosis, as a versatile compound, MDHB deserves more attention to clarify its role in clinical treatment. And more research is needed to clarify the mechanisms that underlie GCs oxidative stress in endometriosis.

### Supplementary Information

**Supplementary Material 1.**

## Data Availability

The datasets used and/or analyzed during the current study are available from the corresponding author on reasonable request.
