# Supplementary material for: Methyl 3,4-dihydroxybenzoate alleviates oxidative damage in granulosa cells by activating Nrf2 antioxidant pathway
Source: J Ovarian Res. 2024 Apr 25;17:87. doi: 10.1186/s13048-024-01412-5 (PMC11044314; doi:10.1186/s13048-024-01412-5)
Supplement: Supplementary file 1 — Supplementary Material 1. [file 13048_2024_1412_MOESM1_ESM.docx]

Table S1 Clinical description of endometriosis and control patients

|  | Control | Endometriosis | P value |
| --- | --- | --- | --- |
| Patients number | 20 | 21 |  |
| Age(years) | 31.22 ± 4.73 | 31.90 ± 3.45 | ＞0.05 |
| BMI (kg/m^2^) | 20.59 ± 2.32 | 22.53 ± 3.27 | ＜0.05 |
| AMH（ng/ml） | 4.15 ± 2.73 | 2.26 ± 1.52 | ＜0.01 |
| Menstrual cycle phase |  |  |  |
| Proliferative | 6 | 12 |  |
| Secretory | 14 | 9 |  |
| Surgical indications | Tubal infertility (13/20)  Male factor(7/20) | Ovarian endometriosis(3/21)  Peritoneal endometriosis(7/21)  Peritoneal and Ovarian endometriosis(4/21)  Peritoneal and deep endometriosis(7/21) |  |
| EM stage | - | Stage I-II :(12/21)  Stage III-IV : (9/21) |  |

AMH: Anti-Mullerian Hormone

EM: endometriosis
